# Effect of Silica Fume Utilization on Structural Build-Up, Mechanical and Dimensional Stability Performance of Fiber-Reinforced 3D Printable Concrete

**DOI:** 10.3390/polym16040556

**Published:** 2024-02-18

**Authors:** Hatice Gizem Şahin, Ali Mardani, Hatice Elif Beytekin

**Affiliations:** 1 Department of Civil Engineering, Faculty of Engineering, Bursa Uludag University, Bursa 16059, Turkey; haticegizemsahin@gmail.com; 2 Department of Architecture, Faculty of Architecture, Bursa Uludag University, Bursa 16059, Turkey; h.elifarslan@gmail.com

**Keywords:** fiber-reinforced 3D printable concrete, polypropylene fiber, silica fume, rheology, thixotropy, drying-shrinkage performance

## Abstract

It is known that 3D printable concrete mixtures can be costly because they contain high dosages of binder and that the drying-shrinkage performance may be adversely affected. Mineral additives and fibers are generally used to control these negative aspects. In this study, the use of silica fume, a natural viscosity modifying admixture, was investigated to improve the rheological and thixotropic behavior of 3D printable concrete mixtures reinforced with polypropylene fiber (FR-3DPC). The effect of increasing the silica fume utilization ratio in FR-3DPC on the compressive strength (CS), flexural strength (FS), and drying-shrinkage (DS) performance of the mixtures was also examined. A total of five FR-3DPC mixtures were produced using silica fume at the rate of 3, 6, 9, and 12% of the cement weight, in addition to the control mixture without silica fume. As a result of the tests, the dynamic yield stress value decreased with the addition of 3% silica fume to the control mixture. However, it was found that the dynamic yield stress and apparent viscosity values of the mixtures increased with the addition of 6, 9, and 12% silica fume. With the increase in the use of silica fume, the CS values of the mixtures were generally affected positively, while the FS and DS behavior were affected negatively.

## 1. Introduction

With current production technologies of Industry 4.0, 3D printing has started to be widely used in the construction sector [1,2,3]. Production of 3D printable concrete (3DPC), which is applied layer by layer without the use of molds [4,5], results in a faster production process compared to traditional concrete [6]. Additionally, various researchers have reported several advantages, including lower labor requirements [7], reduced risk of work accidents [8], higher architectural freedom [9], and partially lower costs [10]. However, it was emphasized that there is no existing standard for 3DPC mixtures [10] and there are constraints such as the need to meet certain fresh-state requirements during production [10]. Serious requirements, especially regarding the rheology of 3DPC, have been noted [11]. While fresh concrete should have relatively lower yield stress and viscosity values during the pumping and extrusion process, it was reported that after extrusion it should have a higher yield stress, viscosity value and an optimum structural build-up rate in order to resist parameters that may cause possible deformation [10,12]. Şahin and Mardani [9] reported that the parameters that can create deformation mentioned are as follows: (i) own weight, (ii) weight of the upper layers, and (iii) high extrusion pressure, respectively.

This is one of the leading comparisons in the development of 3DPC technology, that meets these requirements in 3DPC mixtures in need of rheological care, which is contradictory with comparison to conventional concrete [13,14,15,16,17]. In order to overcome this difficulty, [18] used two parts (i) to accelerate the hydration of fragmented cementitious materials with an interventional process before extrusion and (ii) with the use of a thickening agent. In the first method, it was understood from the literature that nozzle expansions/changes in nozzle shape and cost-increasing additional technologies must be used [19,20,21,22]. In the second method with a thickening agent, the agent is generally added to the mixture with properties such as viscosity-based admixtures [23], silica fume (SF) [24], and nanoparticles [25]. Several studies on the subject are summarized in Table 1.

In studies conducted in the literature, it can be seen that both the thixotropic properties and strength properties of 3DPC mixtures can be positively affected by the addition of these agents [12]. SF and hydroxypropyl methylcellulose were proven to be effective viscosity modifying materials that positively affect the buildability and thixotropic behavior of 3DPC mixtures [26]. The impact of utilizing fly ash and silica fume on the fresh-state performance of 3DPC mixtures was examined in [24]. It was found that the inclusion of fly ash lowers the yield stress and viscosity values of 3DPC combinations, much like in conventional concretes. In contrast, a reversal of this effect was noted when adding silica fume. Furthermore, it was found that the mixture roughness increased. It was highlighted that the ability to deposit more layers without distortion is made possible by an increase in roughness. According to reports, the addition of silica fume improved the mixture printability in terms of buildability parameter and yield stress.

**Table 1:** Examples of studies in the literature on the subject.

Reference	Fiber Type	Supplementary Cementitious Materials (SCM)	SCM Utilization Ratio	Highlights
[27]	polypropylene (PP)	nano silica (NS)	0.005% and 0.01% (of total volume)	The use of NS and PP fiber can decrease the setting time and reduce the collapse of 3DPC. Additionally, the increase in NS and PP fiber usage rate causes the CS values of 3DPC mixtures to decrease.
[28]	-	SF	6%, 10%, and 16% (of cement weight)	With increase of SF dosage, static yield stress (SYS), dynamic yield stress (DYS), and viscosity values of 3D printable foam concrete increase.
[29]	polypropylene (PP)	SF	3% (of cement weight)	The change in PP fiber usage rate significantly affects the viscosity and DYS. The resistance of concrete against cracking is improved by the addition of PP fiber. Concrete using PP fiber can be printed with a low shrinkage when the DYS varies between 250 and 500 Pa and the viscosity varies between 22 and 60 Pa·s.
[30]	steel fiber	SF	5%, 10%, 15%, 20%, and 25% (amount of cement)	3DPC mixtures produced using 10% to 15% SF have the highest fiber-matrix bond, tensile and flexural properties. By image analysis the SF content at these rates leads to lower viscosity and more evenly distributed fibers.

In another study by [31], the effect of nano-silica usage rate (1, 2, and 3%) on the reflocculation value of 3DPC was examined. The highest reflocculation value was measured in the mixture containing 1% NS. With the increase in nano-silica usage rate, the thixotropic behavior of 3DPC was negatively affected and the reflocculation rate decreased. It was reported that this might be due to the increase in the total surface area because of the high specific surface area value of nano-silica and the insufficient fluidizing admixture to disperse the particles in the system. Researchers attempted to impart self-strengthening properties to 3D printable concrete by adding fibers, aiming to eliminate the use of steel in 3D printable buildings [32].

It has been reported by various researchers that in 3D printable concrete mixtures, pozzolan materials such as fly ash, slag, and silica fume are used by substituting them to increase the performance of fresh and hardened properties and especially to reduce the production cost. While the use of pozzolan in 3D printable concrete mixtures positively affects workability and mechanical properties, it causes a decrease in early age strength. It was stated by Marchon et al. [33] that this situation imposes limitations on the use of pozzolans. The complementary material feature and usage rate used in fiber-reinforced 3DPC mixtures (FR-3DPC) seriously affect the rheology and mechanical properties [34]. The use of nanomaterials in fiber-reinforced cementitious systems shortened the hardening time by reducing fluidity [32]. In the literature, it was determined that there are several studies on the effect of SF usage rate change on the fresh- and hardened-state properties of 3DPC mixtures, but conflicting results were obtained among the studies. This study aimed to resolve these contradictions. Additionally, it was understood that limited research had been conducted on the change in silica fume utilization rate, especially in fiber-reinforced 3DPC (FR-3DPC) mixtures, but the rheological properties and thixotropic behavior of 3DPC mixtures were generally not investigated in the studies. In this study, the use of silica fume, a natural viscosity modifying admixture, was investigated in order to improve the rheological properties and thixotropic behavior of 3DPC mixtures reinforced with polypropylene fiber. In addition, the effect of increasing the silica fume usage rate in FR-3DPC on the CS, FS, and DS performance of the mixtures was also examined.

## 2. Material and Method

### 2.1. Materials

F type fly ash in accordance with ASTM C618 Standard [35], silica fume with a diameter of 0.15 µm in accordance with ASTM C1240 Standard [36], and CEM I 42.5R type cement with EN 197-1 Standard [37] were employed as binders in this investigation. The binder materials used within the scope of the study were obtained from Vezirhan Concrete Company in Bilecik province. Table 2 displays components of the binders.

Crushed limestone aggregate with *D*_*max*_ of 1 mm was utilized as the aggregate in the creation of 3DPC mixtures. According to the EN 1097-6 Standard [38], the aggregate’s specific gravity value and water absorption capacity were found to be 2.58 and 0.4, respectively. In the study, polycarboxylate-ether-based high-range water-reducing admixture (HRWR) was used. Table 3 lists some features of the HRWR that was supplied by the manufacturer. It is recommended by the manufacturer to use a ratio of 0.5% to 2% by weight of the total binder in the mixture design for fluid consistencies in all types of concrete classes. This ratio varies depending on the cement, aggregate, mineral additive, and water components used in the concrete mixture design, as well as the desired fresh and hardened concrete properties. For this reason, before determining the usage rate, laboratory tests should be carried out according to the properties expected from fresh and hardened concrete, and the mixing ratio should be determined according to these tests.

Polypropylene fiber with an aspect ratio of 200 was used in all mixtures at the rate of 0.4% of the total volume. It was emphasized by Bentur [39] that in mixtures where 3 mm long short fibers are used, the strength performance of the mixtures may be negatively affected as a result of the increase in local porosity due to the increase in the possibility of multifilament structure formation during mixing.

However, various researchers reported that when longer (12 mm) fibers are used, the strength performance may decrease as a result of curling and clumping [40,41,42,43,44]. On considering the results of the studies in the literature, the length of the fiber used in this study was chosen as 6 mm. Several properties of the polypropylene fiber supplied by the manufacturer are given in Table 4.

### 2.2. Mixtures Ratio

**Figure 1:**
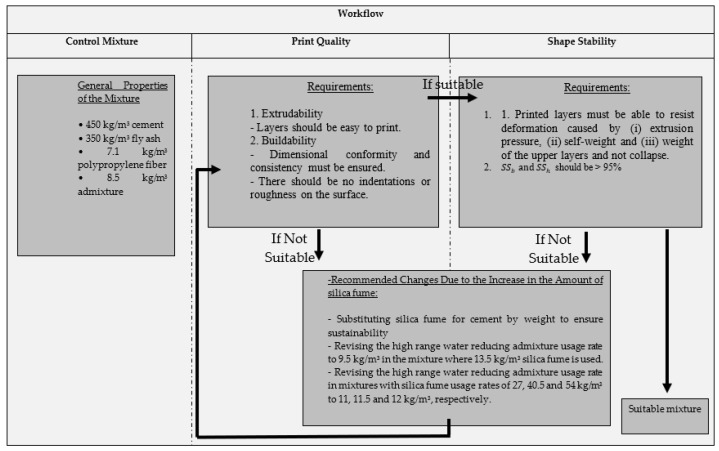
Workflow used within the scope of the study.

**Figure 2:**
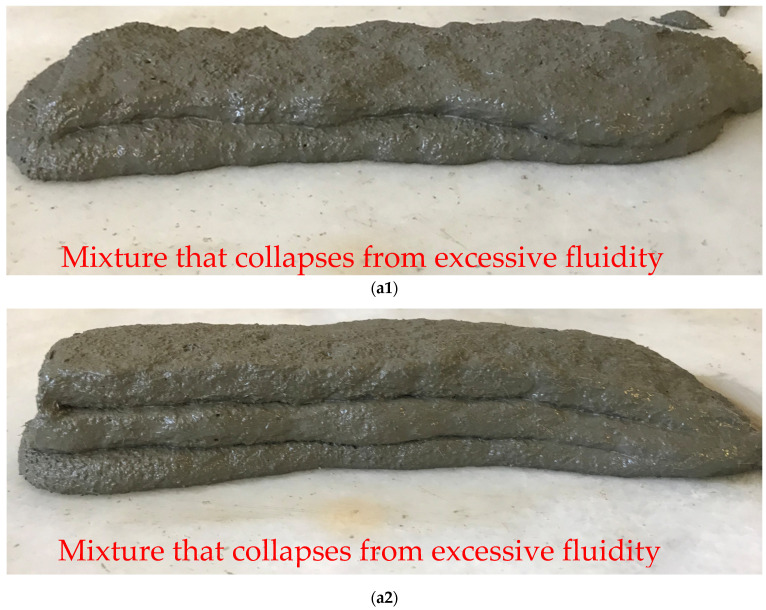

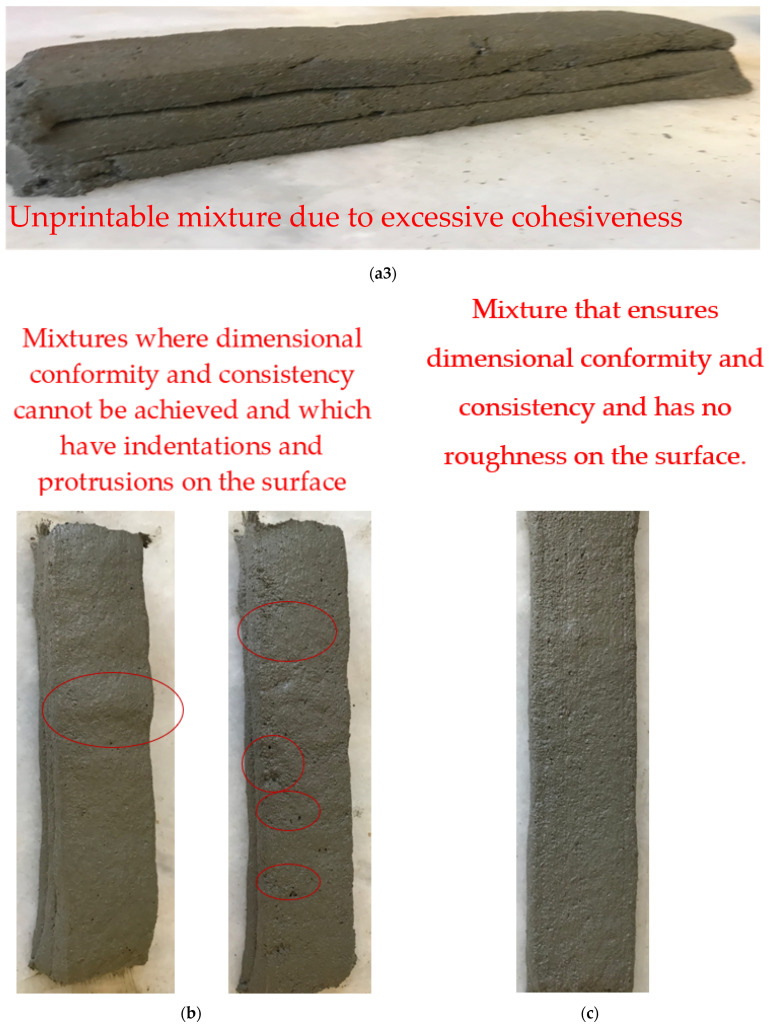

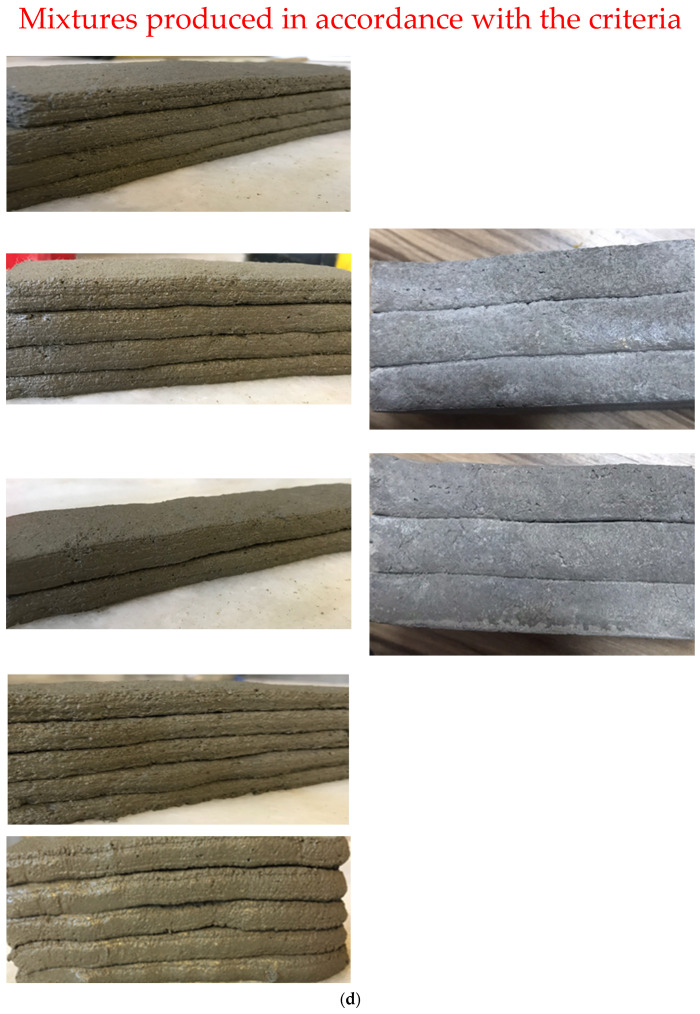
Illustration of mixtures.

The mixing ratios of fiber reinforced 3DPC concretes (FR-3DPC) were determined by taking into account the extrudability, buildability, and shape stability criteria suggested by [45]. In order to understand the criteria mentioned more clearly, the workflow used is shown in Figure 1. A similar workflow was applied by Kazemian et al. [46]. Additionally, images of the produced layers are shown in Figure 2. The print quality parameter was examined under two headings: extrudability and buildability criteria. Mixtures that can be pressed without causing any clogging in the nozzle are considered extrudable [9]. Among extrudable mixtures, mixtures that can be printed as a 5-layer structure and have no roughness on the surface are defined as buildable [9]. For example, in Figure 2(a1,a2), mixtures that cannot produce a 5-layer structure and collapse are shown.

The problem was evaluated as a situation caused by the excessive fluidity of the mixtures, and the amount of HRWR used in the mixtures was revised. Figure 2(a3) shows the situation where the mixture is excessively cohesive, causing clogging in the nozzle, and the buildability criterion is not met because the 5-layer structure cannot be produced. Figure 2b shows mixtures that can be extruded but whose constructability criteria are not met because they have indentations and protrusions on the surface and are not dimensionally suitable. Figure 2c shows an example of a mixture in which dimensional conformity and consistency are achieved and there is no roughness on the surface. The shape stability criterion was investigated in mixtures selected as appropriate in terms of print quality (2d). Mixtures with a shape-stability value greater than 95%, calculated according to Equations (1) and (2), are considered to be suitable in terms of 3D printability criteria. Mixtures suitable for shape stability are shown in Figure 2d. In order to determine the shape stability of the printed mortar mixtures, a 5-layer structure was printed for each mixture. Since it is known that the shape stability is affected by the characteristics of the injection gun or printer used, as well as the mixture properties, care was taken to ensure the same device and environmental conditions were used for the shape stability of each mixture. Past experiences and visual reasoning results were considered in determining this ratio. Since the nozzle width used in the study was 42 mm, it was accepted that the shape-stability criterion was met if the layer width was between 40 and 42 mm.

Similar assumptions were made by other researchers [46,47]. In a study by Saruhan et al. [47], it was reported that the shape-retention ability of 3D printable concrete mixtures was calculated by dividing the substrate filament width by the extruder nozzle width. Researchers have stated that the buildability of a mortar mixture increases as the shape retention coefficient approaches 1. It is emphasized that it should be noted that this method actually focuses only on shape retention ability.

(1) $${SS}_{b}(\%)=\frac{b_{layer}}{b_{nozzle}}\cdot 100$$

(2) $${SS}_{h}(\%)=\frac{h_{layer}}{h_{nozzle}}\cdot 100$$

where,

SS_b_; shape stability of layer width, SS_h_; shape stability of layer height (%), $b_{layer}$; layer width after extrusion (mm) and $b_{nozzle}$ represents the nozzle width (42 mm), $h_{layer}$; layer height after extrusion (mm) and $h_{nozzle}$ represents the nozzle height (15 mm).

450 kg cement, 350 kg fly ash, 1024.2 kg aggregate, 7.1 kg polypropylene fiber and 8.5 kg HRWR were used in 1 m^3^ FR-3DPC mixture, which does not contain silica fume and where the mentioned criteria are met. FR-3DPC combinations were generated in five different series by adding silica fume to the control mixture at varied rates of 3, 6, 9, and 12% of the cement weight. This was done to investigate the impact of silica fume usage and usage rate on several properties of the mixtures in fresh and hardened states. The amount of material used in the production of 1 m^3^ FR-3DPC mixtures is shown in Table 5. The naming of the mixtures is made according to the silica fume usage rate. For example, the mixture containing 6% silica fume is named SF-6%.

The w/b ratio was kept constant at 0.35. In order to meet the compliance criteria, HRWR was added at different rates. HRWR requirement in mixtures increases with the addition of silica fume. HRWR requirement of the SF-12% mixture containing 12% silica fume increased by 71% compared to the control mixture. This was due to the increase in the total surface area of the matrix with the addition of silica fume.

### 2.3. Method

#### 2.3.1. Preparation of Mixtures

The mixtures were prepared in three different stages. In the first stage, binder materials (cement, fly ash, and silica fume), fine aggregate, and fiber were mixed at 62.5 rpm for 1 min. In the 2nd stage, water and HRWR were added to the mixture and mixed at 62.5 rpm for 1 min. In the 3rd stage, the mixture preparation process was completed by mixing the mixture at 125 rpm for 2 min. Three samples were prepared for each experiment. The specimens were prepared by injection gun extrusion with the nozzle output for mortars being 42 mm wide and 15 mm high [45]. The injection tool has a cylindrical container (Ø65 × 300 mm). FR-3DPC samples were printed using an injection gun at a printing speed of approximately 5 mm/s. Similar devices and procedures have been used by many researchers [47,48]. The produced samples were kept in a curing cabinet with a temperature of 20 ± 1 °C and a relative humidity of 90 ± 5% immediately after casting. After 24 h, the sample was subjected to water cure of 20 ± 1 °C until the day of the experiment.

#### 2.3.2. Rheological Procedure

**Figure 3:**
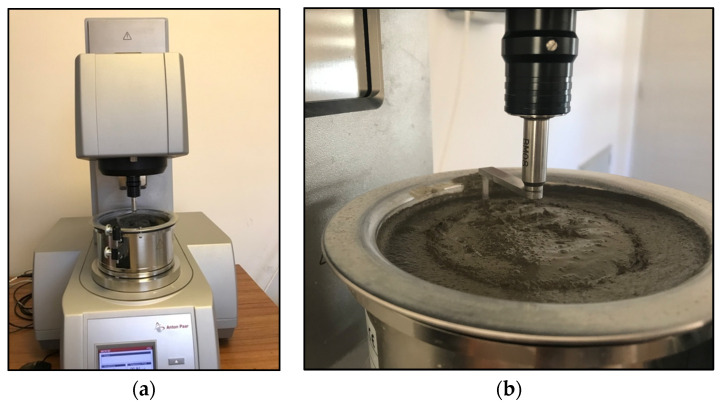
Rheometer device used for rheological measurements of FR-3DPC mixtures and (**b**) sample mixture.

**Figure 4:**
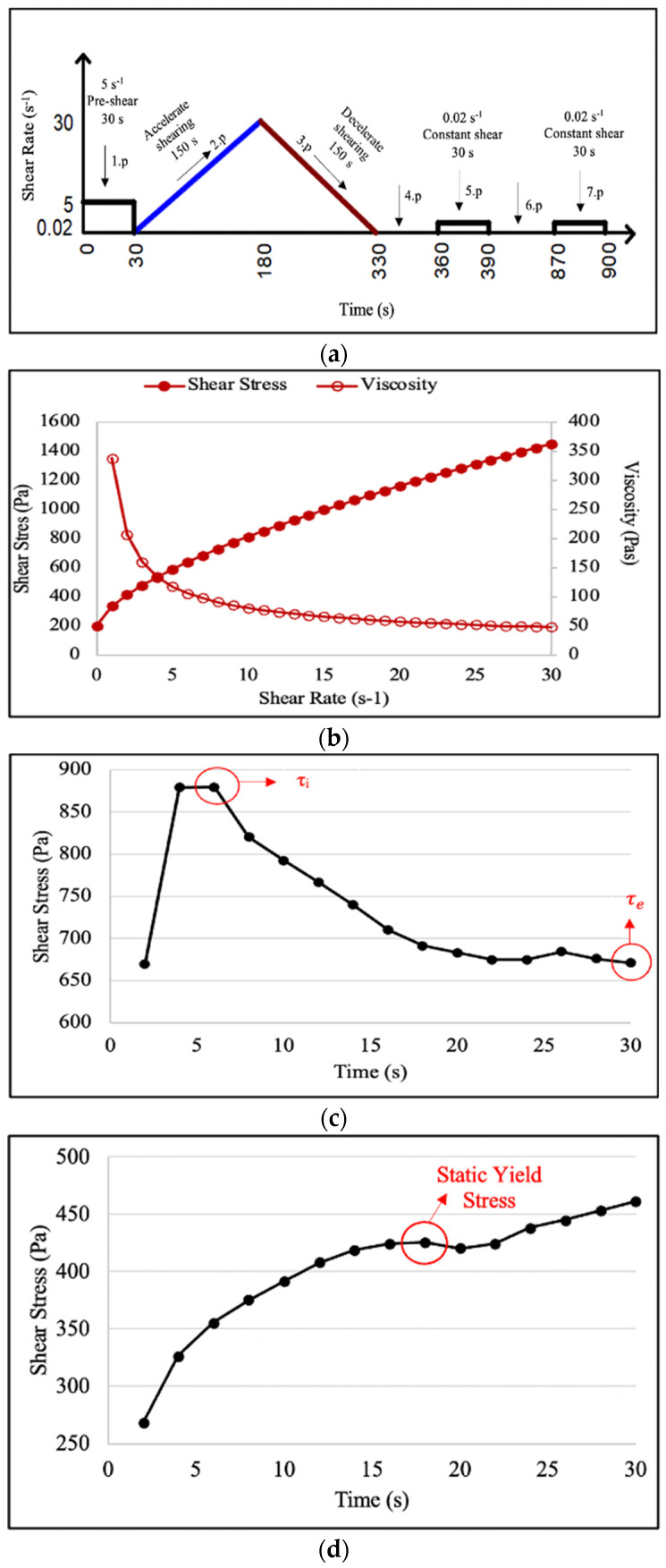
(**a**) Rheological measurement process. (**b**) Determination of DYS and viscosity values from the 3rd period. (**c**) Determination of τ_i_ and τ_e_ values required to calculate the I_thix_ value from the 5th and 7th periods. (**d**) Determination of the SYS required to calculate the A_thix_ value from the 5th and 7th periods.

Rheological measurements of the mixtures were carried out immediately after production, without waiting. For this purpose, the MCR52-Anton Paar rheometer (Figure 3a) with a ball diameter of 8 mm was used. Figure 3b shows the image of the mixture subjected to rheology testing. A rheological measurement process consisting of 7 periods shown in Figure 4 was applied to determine the rheological parameters. The rheological measurement method used was created by modifying two different rheological measurement methods suggested by Mardani-Aghabaglou [49] and Yao et al. [50].

1st Period: This period was applied to eliminate shear history during mixing in the mixer. With a constant deformation rate of 5 s^−1^, the ball was rotated in the mixture for 30 s.

2nd Period: This period was used to create the output part of the flow curve. The deformation rate was increased from 0 to 30 s^−1^. Measurements were taken every 5 s for a total of 150 s.

3rd Period: This period was made to create the downward part of the flow curve. Shear rate is reduced from 30 to 0 s^−1^. Measurements were taken every 5 s for a total of 150 s. Dynamic yield stress (DYS) and apparent viscosity values of FR-3DPC mixtures were obtained from this period. To determine the DYS values, the flow curve was drawn for each mixture, taking into account the raw data from the 3rd period obtained from the rheometer. As a result of analyzing the flow curve data with the help of the Herschel–Bulkley model, DYS and final viscosity values were calculated for each mixture. The values obtained from the model were taken as DYS values. The point where each curve intersects the y-axis is defined as the “DYS”. Shear stress–shear rate and viscosity–shear rate graphs for each mixture were obtained in Equation (3), using the raw data determined in the 3rd period. In this model, the viscosity value is measured instantly. However, when the viscosity curve is taken into consideration, it is seen that the viscosity value does not change after a certain shear rate. This value was taken as the final viscosity value of that mixture. Figure 4a shows the shear stress–shear rate and viscosity–deformation rate graphs of the FR-3DPC mixture.

(3) $$\tau =\tau_{0}+b{\dot{\gamma }}^{p}$$

Here, τ: the shear stress (Pa), $\tau_{0}$: yield stress (Pa), b: the Herschel–Bulkley consistency coefficient, $\dot{\gamma }$: shear rate (s^−1^) and p: the Herschel–Bulkley index.

4th Period: This period was applied to recover the FR-3DPC mixtures before static measurement. The mixtures were kept for 30 s without being exposed to any shear rate.

5th Period: In this period, moment measurements were taken every 2 s for a total of 15 times for 30 s with a constant shear rate of the mixture (0.02 s^−1^). During this period, the SYS of the material was measured.

6th Period: In this period, the mixtures were kept for 480 s without being exposed to any deformation rate in order to measure the structural build-up rate.

7th Period: In this period, moment measurements were taken every 2 s for a total of 15 times for 30 s with a constant shear rate of the mixture (0.02 s^−1^). During this period, the SYS was measured.

#### 2.3.3. Thixotropic Measurement

Three different methods, one dynamic and two static, were used to determine the thixotropic properties of FR-3DPC.

In method 1, which is the dynamic approach, the thixotropic behavior of the mixtures was measured in accordance with the method proposed by [11]. In this study, in the method called dynamic structural build-up (D-SBU), the thixotropic properties of mixtures were examined by measuring them in the 3rd and 2nd periods (Equation (4)):

(4) $$D-SBU=\frac{\tau_{3.p}}{t_{2.p}}$$

Here,

D−SBU dynamic structural build-up, $\tau_{3.p}$ dynamic yield stress (Pa) obtained from the 3rd period. and $\tau_{2.p}$ dynamic yield stress obtained from period 2 (Pa).

In order to perform the second method, which is the static approach, the mixture was left for 30 s immediately after the dynamic measurements. Then, initial static measurements were carried out at a constant shear rate of 0.02 s^−1^ for 30 s. Immediately after waiting for 480 s, the final static measurements of the mixture were carried out at a constant shear rate of 0.02 s^−1^ for 30 s. Using the SYS values obtained as a result of this test, the structural build-up (A_thix_) of the mixture was determined with the help of Equation (5). Figure 4b shows the procedure for determining the SYS required to calculate the A_thix_ value. Accordingly, the highest shear stress obtained from the shear stress–time graph of the mixture was recorded as the SYS of the mixture. The A_thix_ value of the FR-3DPC mixture was calculated using the SYS obtained from the data in the 5th and 7th periods.

(5) $$A_{thix}=\frac{\tau_{s,f}-\tau_{s,i}}{t_{d}}$$

Here,

$A_{thix}$ structural build-up (Pa/s), $\tau_{s,f}$ SYS value (Pa) obtained from the 7th period, $\tau_{s,i}$ SYS value (Pa) obtained from the 5th period, and $t_{d}$ represents the duration time (480 s).

As is known, the typical shear-stress behavior of cementitious systems under a constant shear rate begins with a rapid increase to a maximum value (τ_i_), followed by a gradual decrease until it reaches the equilibrium value (τ_e_). It was reported by Qian and Kawashima [51] that this situation is associated with thixotropy. A number of theories have been proposed to assess thixotropy and explain the exponential decline of shear stress over time. In this study, in the 3rd method, which is the static approach, the thixotropic behavior of the mixtures was evaluated with the thixotropic index (I_thix_) value calculated by Equation (6). Figure 4c shows the procedure for determining τ_i_ and τ_e_ values required to calculate the I_thix_ value. Accordingly, the highest shear stress obtained from the shear stress–time graph of the mixture was recorded as τ_i_ and the lowest shear stress was recorded as τ_e_. The I_thix_ value of the FR-3DPC mixture was calculated using the τ_i_ and τ_e_ values obtained from the data in the 5th and 7th periods:

(6) $$I_{thix}=\frac{\tau_{i}}{\tau_{e}}$$

where,

I_thix_: thixotropic index, $\tau_{i}$: maximum shear stress needed to initiate flow, and $\tau_{e}$: steady-state flow.

#### 2.3.4. Hardened State Properties

The 7- and 28-day CS, three-point FS and DS performance properties of FR-3DPC mixtures were measured as hardened state properties. The strength performance of the samples was determined by making some changes in the dimensions and loading directions specified in the EN 196-1 Standard [52]. For this purpose, the samples were loaded on 40 mm × 40 mm surfaces and their CS was determined. Also, the FS of the printed samples was determined by performing a three-point bending test on 40 mm × 40 mm × 160 mm prism samples.

Prismatic samples of 25 mm × 25 mm × 285 mm were produced to examine the DS behavior of the mixtures. Unlike the curing condition in other tests, the samples were kept in a cabin with a relative humidity of 55% after 24 h of water cure. The length change of prismatic samples was calculated according to ASTM C 596-01 as shown in Equation (7):

(7) $$S=\frac{L_{1}-L}{L_{0}}\cdot 100$$

where,

S; percent shrinkage of the sample, L_1_; initial measurement value after being removed from the curing pool, L; refers to the periodic measurement value and L_0_ refers to the effective measurement length.

## 3. Results and Discussion

### 3.1. Setting Time of Mixtures

**Figure 5:**
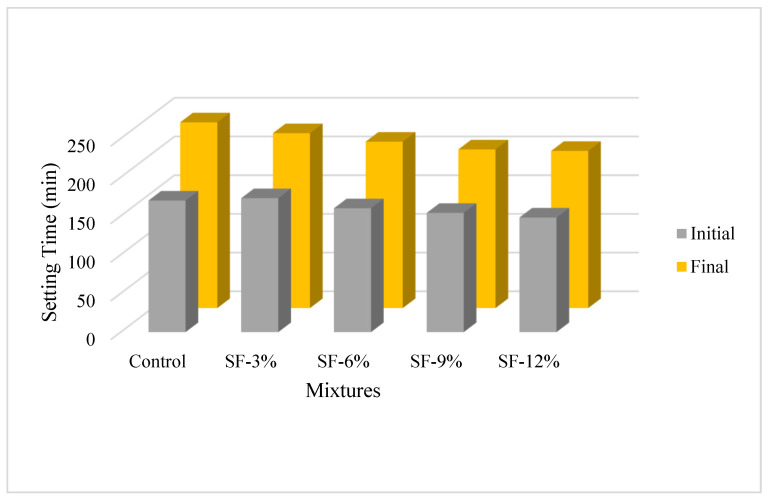
Setting time of paste mixtures.

The initial and final setting times of the paste mixtures produced within the scope of the study are shown in Figure 5. As can be clearly seen from the figure, it is understood that adding 3, 6, 9, and 12% silica fume to the control mixture generally causes the initial and finish time to decrease. It was determined that the decrease in setting time was more obvious due to the increase in the silica fume usage rate. It was also determined that the decrease in the initial and final setting time of the mixtures was highest in the SF-12% mixture, and the decrease occurred by 13% and 16%, respectively. It is thought that this situation is due to the increase in the rate of water consumption due to the increase in the heat of hydration caused by the increase in the fineness of the silica fume. Similar statements were also reported by Şahin et al. [53].

### 3.2. Rheological Properties and Thixotropic Behavior

DYS, apparent viscosity, and structural build-up values are shown in Table 6 and Table 7. As explained before, the DYS and viscosity values of the mixtures were calculated with the data obtained from the 3rd period of the rheological measurement process. The DYS value decreased by 25% with the addition of 3% silica fume to the control mixture. Similarly, the thixotropic behavior of the SF-3% mixture was found to decrease by 27, 49, 14, and 11% according to the D-SBU, A_thix_, I_thix_ in the 5th period and I_thix_ in the 7th period methods, respectively. It is thought that this is due to the fact that when energy is applied to the mixture during measurement, the spherical silica fume particles act as ball bearings and lubricate the mixture, providing more mobility than mixtures with similar slump values. Similar results were expressed by [54].

It was determined that by adding 6, 9, and 12% silica fume to the control mixture, the DYS and apparent viscosity values of the mixtures increased by 25–38%, 40–175%, 233–139%, 0.5–127%, respectively. In addition, the thixotropic behavior of FR-3DPC mixtures generally improves (increases) with the increase in silica fume content in mixtures with silica fume addition above 3%. The highest increase in thixotropic behavior compared to the control mixture was obtained in the SF-6% and SF-9% mixture and the A_thix_ method. In a similar study involving SF by [55], it was reported that SF has an optimum usage rate on its rheological properties. It was declared by the authors that substituting up to 10% of cement with SF increases the buildability of 3D printable concrete mixtures by providing higher SYS without affecting their extrudability. It was emphasized that silica fume addition has two different effect mechanisms on the flow performance of cementitious systems [56]. On the one hand, silica fume improves the flow performance of mixtures by acting as a ball bearing due to its spherical shape. However, on the other hand, since it is very thin, it increases the surface area in the matrix, causing an increase in internal surface forces [57]. It was reported that this situation causes the concrete to become more cohesive and the DYS and viscosity value of the mixture to increase [58]. It was also understood from the experimental results that the first mechanism was more dominant when silica fume was used at low dosage, and the second mechanism was more dominant when it was used at a higher rate.

It was emphasized in the literature by various researchers [59,60] that concretes produced using SF are stickier and more difficult to segregate than normal concrete, even in flowable concretes. The reduction in segregation tendency is known to be an important parameter for mixtures with high fluidity, such as 3D printable concrete mixtures.

It was determined that pumpability and extrusion properties are positively affected by adding a small amount of SF to the 3D printable concrete mixture. In a study by Inozemtcev [61], the use of SF in 3D printable concrete mixtures was found not only to increase the CS and ultimate working capacity by 53% and 88%, respectively, but also to allow the creation of a highly mobile structure. In another study by [62], it was determined that the use of 10% SF in 3D printable geopolymer concrete mixtures improved structural build-up rates that would meet the rheological requirements of 3D printing in the flocculation and polycondensation stages. In another study by [63], the effect of SF usage rate change (4, 8, 12, and 16%) on the rheological properties and interlayer bonding strength of 3D printable concrete mixtures was examined. With the increase in SF usage rate, the initial yield stress value of the mixtures increased, and the thixotropic behavior was positively affected. However, increase in the use of SF from 12% to 16% did not have a significant effect on the rheological properties of the mixtures. Additionally, it was emphasized by the authors that with the increase in the use of SF, the interlayer adherence increased by 40% due to the yield stress of 3DPC not increasing too much. It was emphasized that, in cases where the workability of the mixture is not negatively affected by the addition of SF, the interlayer adherence is positively affected.

The correlation between four different thixotropy measurement methods used in this study is summarized in Table 8. When the data in Table 8 are examined, it is understood that the methods with the highest r value (0.94) are A_thix_-I_thix_ in the 7th period.

**Figure 6:**
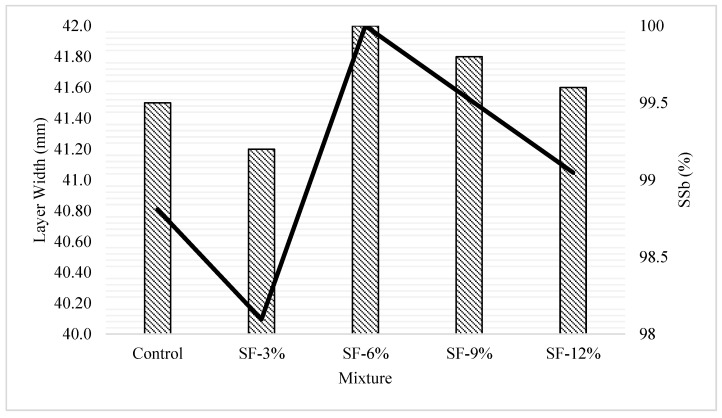

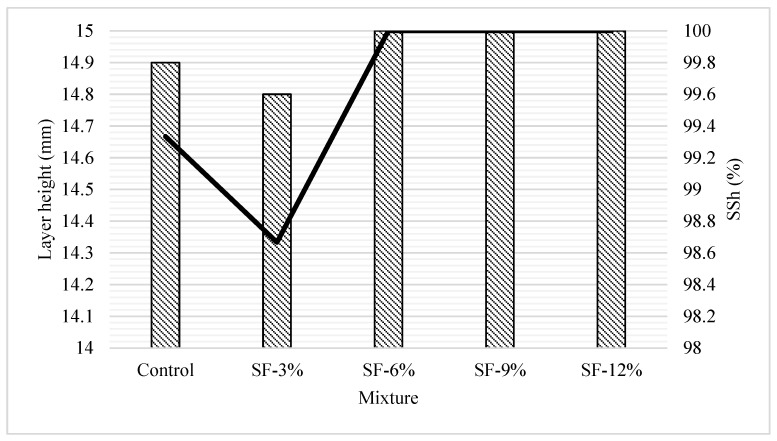
Layer width/layer height and shape stability values of the mixtures.

The layer width-height and shape-stability values obtained depending on the silica fume usage rate in the mixtures produced within the scope of the study are shown in Figure 6. As can be seen from the figure, all mixtures produced are considered suitable in terms of shape stability. It was determined that the mixtures with the highest SS_b_ and SS_h_ values were SF-6%, SF-9%, and SF-12%, respectively, and the mixture with the lowest value was SF-3%. Considering that the SF-3% mixture is the mixture with the lowest dynamic yield stress, D-SBU, A_thix_, and I_thix_ values, it is understood that the rheological properties and shape stability of the mixtures are highly interconnected. In this case, as a result of the materials used and the tests performed within the scope of this study, it was determined that the lower limit of the values of DYS, D-SBU, A_thix_, and I_thix_ in the 5th period and I_thix_ in the 7th period should be 107.11, 0.75, 0.23, 1.25, and 1.15, respectively, in terms of shape stability. In addition, it was measured that the SF-9% mixture, which had the highest values in terms of DYS, D-SBU, A_thix_, and I_thix_ in the 5th period and I_thix_ in the 7th period, had a SS_b_ value of 99.5%. In this case, it was determined that the optimum value in terms of rheological properties and thixotropic behavior belongs to the SF-6% mixture, and the shape stabilities of mixtures with a value above this value are negatively affected. When the relationship between shape stability and rheological properties and thixotropic behavior was examined (Table 9), it was understood that SS_b_ was most affected by the viscosity of the mixture and least affected by the D-SBU value. It was seen that SS_h_ was most affected by the I_thix_ in the 5th period value and least affected by the D-SBU value.

### 3.3. Hardened State Properties

#### 3.3.1. Compressive Strength

**Figure 7:**
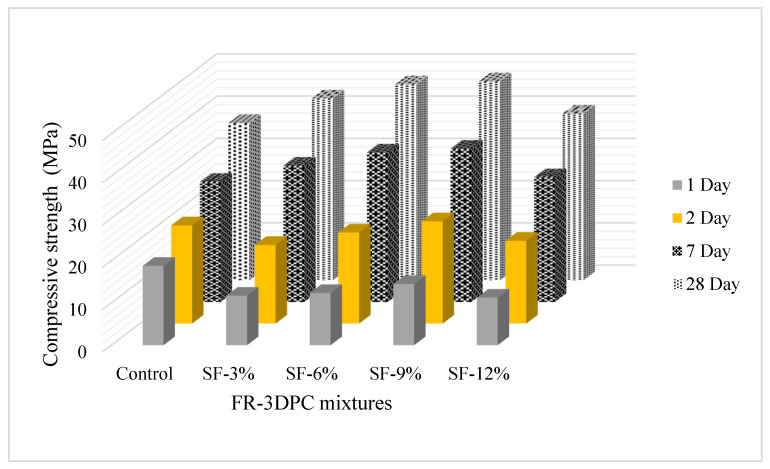
The 7- and 28-day CS results of FR-3DPC mixtures.

The 1, 2, 7, and 28-day CS results are shown in Figure 7. According to the 1- and 2-day compressive strength of the produced mixtures, it was determined that the use of silica fume generally caused the compressive strength value of the mixtures to decrease compared to the control mixture. Similarly, in a study conducted by Özen et al. [64], it was found that the use of silica fume caused the 1-day strength of the mixtures to decrease. It was reported that this situation was due to the decrease in the amount of cement. CS performance was positively affected by the addition of silica fume to the control mixture at 7 and 28 days. This behavior became even more pronounced with an increase in the silica fume usage rate up to 9%. It was seen that by adding 3, 6, 9, and 12% silica fume to the control mixture, the 28-day CS values of the mixtures increased by 15, 25, 27, and 6%, respectively. The strength performance in cementitious systems is positively affected due to the physico-chemical effect created by the addition of SF [65]. It was reported by various researchers that the use of SF increases the homogeneity in the cement paste and reduces the number of large pores due to the higher fineness value (physical effect) [66]. In addition, it increases the strength performance by creating a denser structure due to the increasing C-S-H phase as a result of the chemical pozzolanic reaction [67]. The increased strength performance as a result of the addition of SF is due to the stronger interfacial transition zone (ITZ) in mixtures with SF [68,69]. It was emphasized that this enhanced bond results from the transformation of CH, which tends to form on the surface of aggregate particles, into C-S-H in the presence of reactive silica. In a study conducted by Siddique [70], it was emphasized that the use of SF increased the strength value of the system by strengthening the cement paste–aggregate bond and creating a less porous and more homogeneous microstructure in the interface region. In another study [71] in which the effect of the use of SF on the strength properties of fibrous concrete mixtures was investigated, it was reported that the strength performance increased with the use of fiber. It was emphasized that this situation was due to the more homogeneous distribution of the fibers in the matrix with the addition of SF. Additionally, it was reported by many researchers that fiber-matrix adrenaline increases with the addition of SF, especially in fiber-containing mixtures [72].

It was interpreted from the results that there was an optimum silica fume usage rate in terms of the strength performance of the mixtures. In this study, it was previously emphasized that the optimum usage rate is 9%. This is due to the fact that the length change due to DS is also high in mixtures with high amounts of silica fume. Similarly, it was declared by many researchers that the optimum usage rate of SF for conventional concrete is 10% [56]. It was emphasized that this situation is due to the high fineness value of SF and the formation of a more porous structure as a result of increasing the risk of agglomeration formation by increasing the water requirement when used above the optimum rate [56,57]. It was reported that the use of SF significantly increases the height change caused by DS at the early ages [73]. In a study conducted by [55], the effect of changing the SF usage rate on the CS of 3D printable concrete mixtures was examined. It was determined that the CS values of mixtures containing 5% and 10% SF were lower than the control mixture. Researchers stated that fly ash is the parameter that determines the CS of the samples in mixtures containing SF and fly ash [74].

#### 3.3.2. Flexural Strength

**Figure 8:**
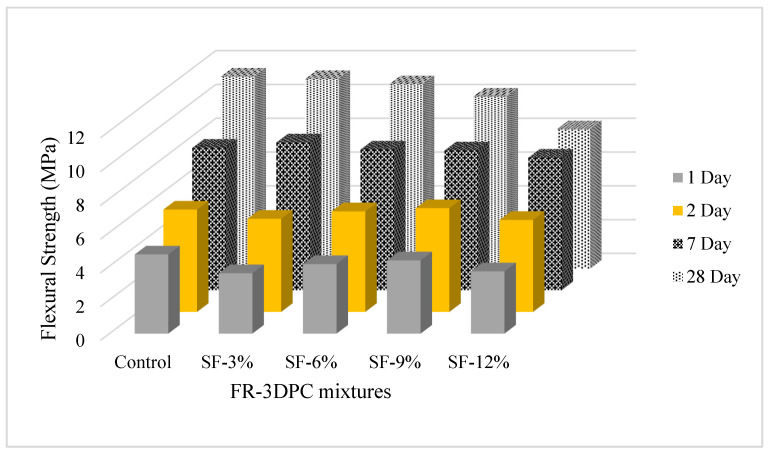
The 7- and 28-day FS results of FR-3DPC mixtures.

The 1, 2, 7, and 28-day FS results of the mixtures are shown in Figure 8. It is understood from the figure that, contrary to the trend in CS values, the change in silica fume usage rate has a different effect on the FS value of FR-3DPC. It is thought that this is due to the fact that the pressure and flexural test setups are different, and the FS is more sensitive to microcracks caused by shrinkage. It was found that the addition of silica fume to the control mixture did not have a significant effect on the 7-day FS of the mixtures, while the flexural strength of the 1- and 2-day mixtures generally caused a decrease. However, the 28-day FS of the mixtures was negatively affected by increasing the silica fume usage rate. It was measured that by adding 3, 6, 9, and 12% silica fume to the control mixture, the 28-day FS values decreased by 2, 4, 11, and 28%, respectively. This is due to the increase in length change due to shrinkage during early drying because of the high fineness value of silica fume. DS test results of the mixtures proved this claim. In a study conducted by [75], it was determined that the tensile strength of the mixtures was not significantly affected by substituting SF at 0, 5, 10, 15, 20, and 25% of the cement weight. However, in another study conducted by [76], it was seen that the increase in the use of SF increased both the CS and FS of cementitious systems. Similarly, in a similar study conducted by [77], it was found that the tensile strength of cementitious systems was not affected by 8% SF substitution. However, researchers reported that the pozzolanic and filling effect of SF improves especially the CS properties of cementitious systems.

#### 3.3.3. Drying-Shrinkage Performance

**Figure 9:**
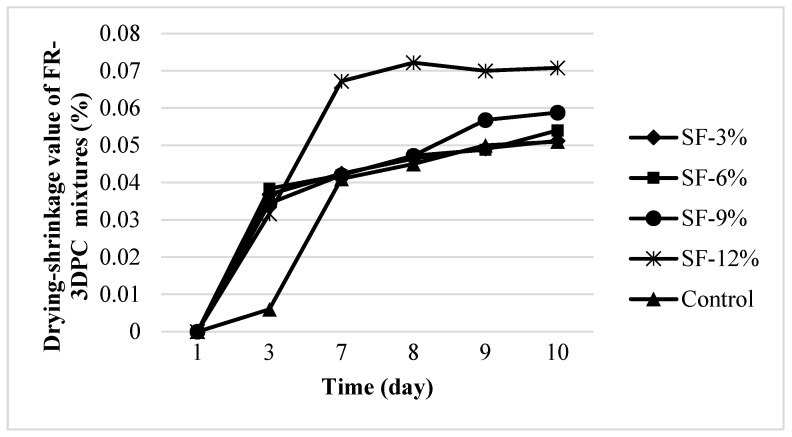
Length-change performance of FR-3DPC mixtures due to drying-shrinkage.

Cracks in cementitious systems generally develop over time due to various reasons such as plastic shrinkage during pre-hardening as well as DS in hardened concrete. In future time, these cracks negatively affect the permeability properties of concrete, causing a decrease in durability performance [78]. The DS performance of FR-3DPC mixtures is shown in Figure 9. Not surprisingly, while the shrinkage-induced length-change increase rate of the mixtures was high in the first days, this rate decreased over time. The length change due to DS increased with the addition of silica fume to the control mixture, regardless of the silica fume usage rate. It was determined that by adding 3, 6, 9, and 12% silica fume to the control mixture, the length change due to DS increased by 0.4, 5, 15, and 38%, respectively. Shrinkage in cementitious systems is a physicochemical process, and it was emphasized that hydration reactions begin with the hydration of the mixing water [43]. Capillary voids form within the cement paste as it begins to harden. After this stage, hydration reactions continue with the recall of pore water. A physical effect dominates the shrinkage of spaces that lose water. On the other hand, it was reported that structural shrinkage accelerates during the period when the heat released during hydration development is highest [43]. It was found that silica fume is effective in early DS due to its very high pozzolanic activity and fineness mechanism, and that the increase in the amount of use negatively affects the DS performance. As it is known, the increase in the amount of silica fume usage seriously affects the DS behavior of cementitious systems. However, this effect is in complex balance. In the literature, it was understood that the effect of SF use on the performance of concrete varies depending on the amount of use, the type and amount of cement used, the amount of fiber use, the amount of aggregate, and the chemical additives employed [72]. When previous studies were examined, it was seen that SF was generally used to improve the performance of cementitious systems strengthened using polypropylene [56]. In the study conducted by [68], cement was substituted with 10% SF to provide high strength and durability properties in cementitious systems where polypropylene and steel fiber were used as a hybrid. It was found that the use of polypropylene fiber and SF causes significant decreases in the water absorption of concrete. It is thought that this situation may also positively affect the DS values.

## 4. Conclusions

As a result of the experiments, the following conclusions were obtained:

1. The measured DYS value and thixotropic behavior decreased with the addition of 3% silica fume to the control mixture.
2. DYS and apparent viscosity values of the mixtures generally increased with the addition of 6, 9, and 12% silica fume to the control mixture.
3. The thixotropic behavior of FR-3DPC mixtures generally improved with the increase in silica fume content.
4. The highest increase in thixotropic behavior compared to the control mixture was obtained in the SF-6% and SF-9% mixture and the A_thix_ method.
5. It was found that the methods with the highest Multiple R value (0.94) were A_thix_-I_thix_ in the 7th period.
6. It was determined that the lower limit of the values of DYS, D-SBU, A_thix_, and _Ithix_ in the 5th period and I_thix_ in the 7th period should be 107.11, 0.75, 0.23, 1.25, and 1.15, respectively, in terms of shape stability.
7. It was apparent that adding silica fume generally caused the initial and finish time to decrease.
8. It was found that the use of silica fume generally caused a decrease in the 1- and 2-day compressive strength of the mixtures.
9. It was shown that the addition of silica fume to the control mixture generally caused a decrease in the 1- and 2-day flexural strength of the mixtures.
10. The 7–28-day compressive strength values of the mixtures increased by adding 3, 6, 9, and 12% silica fume to the control mixture.
11. The 28-day flexural strength of the mixtures was negatively affected by increasing the silica fume utilization ratio.
12. Adding 3, 6, 9, and 12% silica fume to the control mixture caused the length change due to drying-shrinkage to increase by 0.4, 5, 15, and 38%, respectively.

## Figures and Tables

**Table 2:** Components of the binders.

Oxides (%)		Cement	Fly Ash	Silica Fume
SiO_2_		18.00	58.79	79.13
Al_2_O_3_		4.75	22.51	0.71
Fe_2_O_3_		3.58	7.89	0.33
CaO		63.00	3.70	0.21
MgO		1.40	2.18	7.73
Na_2_O + 0.658 K_2_O		0.70	1.93	
SO_3_		3.11	0.29	0.97
Specific gravity		3.06	2.35	2.10
Specific surface (cm^2^/g)		3441	4000	18,000
Setting Time (min)	Initial	170	-	-
	Final	240	-	-
Compressive Strength (MPa)	7-Day	42.80	-	-
	28-Day	51.80	-	-
Pozzolanic activity index (%)	28-Day		77.70	100
	90-Day		92.50	132

**Table 3:** HRWR properties.

Density (g/cm^3^)	Solid Matter (%)	pH	Na_2_O (%)	Chlorine (%)
1.060	32	2–5	<10	<0.1

**Table 4:** Polypropylene fiber properties.

Aspect Ratio (Length/Diameter)	Fiber Length (mm)	Modulus of Elasticity (MPa)	Tensile Strength (MPa)	Specific Weight	Surface Area (m^2^/kg)
200	6	4861	500	0.91	140

**Table 5:** Amounts of component used in the production of 1 m^3^ FR-3DPC (kg/m^3^).

Mix	Cement	Fly Ash	Silica Fume	Aggregate	Polypropylene Fiber	HRWR	w/b
Control	450	350	-	1024.2	7.1	8.5	0.35
SF-3%	436.5		13.5	1016.6		9.5	
SF-6%	423		27	1007.7		11.0	
SF-9%	409.5		40.5	1001.3		11.5	
SF-12%	396		54	994.9		12	

**Table 6:** Rheological properties of FR-3DPC mixtures.

Mixture	DYS in the 2nd Period (Pa)	DYS in the 3rd Period (Pa)	Maximum SYS in the 5th Period (Pa)	Equilibrium SYS in the 5th Period (Pa)	Maximum SYS in the 7th Period (Pa)	Equilibrium SYS in the 7th Period (Pa)	Viscosity in the 3rd Period (Pa·s)
Control	138.7	143.3	420.06	289.95	641.62	494.88	17.62
SF-3%	142.28	107.11	257.36	205.7	369.12	321.42	24.44
SF-6%	258.14	201.97	573.19	363.84	1055.7	805.26	48.38
SF-9%	306.1	476.63	999.69	615.01	1865.9	1256	42.18
SF-12%	139.9	144.15	461.55	268.53	879.68	671.24	40.06

**Table 7:** Thixotropic behavior of FR-3DPC mixtures.

Mixture	D-SBU	A_thix_ (Pa/s)	I_thix_ in the 5th Period (Pa)	I_thix_ in the 7th Period (Pa)
Control	1.03	0.46	1.45	1.30
SF-3%	0.75	0.23	1.25	1.15
SF-6%	0.78	1.01	1.58	1.31
SF-9%	1.56	1.80	1.63	1.49
SF-12%	1.03	0.87	1.72	1.31

**Table 8:** Relationship between thixotropy measurement methods.

Method	D-SBU-A_thix_	D-SBU-I_thix_ in the 5th Period	D-SBU-I_thix_ in the 7th Period	A_thix_-I_thix_ in the 5th Period	A_thix_-I_thix_ in the 7th Period	I_thix_ in the 5th Period-I_thix_ in the 7th Period
Multiple R	0.81	0.50	0.89	0.70	0.94	0.73
R^2^	0.66	0.25	0.79	0.49	0.89	0.53
Standard Error	0.22	0.32	0.17	0.50	0.23	0.14

**Table 9:** Relationship between rheological properties and shape stability.

Method	SS_b_-DYS in the 3rd Period	SS_b_-Viscosity in the 3rd Period	SS_b_-D-SBU	SS_b_-A_thix_	SS_b_-I_thix_ in the 5th Period	SS_b_-I_thix_ in the 7th Period
Multiple R	0.53	0.82	0.30	0.73	0.72	0.69
R Square	0.29	0.67	0.09	0.54	0.52	0.48
Adjusted R Square	0.05	0.55	−0.21	0.39	0.37	0.30
**Method**	**SS_h_-DYS in the 3rd Period**	**SS_h_-Viscosity in the 3rd Period**	**SS_h_-D-SBU**	**SS_h_-A_thix_**	**SS_h_-I_thix_ in the 5th period**	**SS_h_-I_thix_ in the 7th period**
Multiple R	0.53	0.80	0.48	0.79	0.96	0.78
R Square	0.28	0.64	0.23	0.62	0.92	0.61
Adjusted R Square	0.04	0.52	−0.02	0.49	0.90	0.48

## Data Availability

Data are contained within the article.
